# Models for Cell-Free Synthetic Biology: Make Prototyping Easier, Better, and Faster

**DOI:** 10.3389/fbioe.2018.00182

**Published:** 2018-11-29

**Authors:** Mathilde Koch, Jean-Loup Faulon, Olivier Borkowski

**Affiliations:** ^1^Micalis Institute, INRA, AgroParisTech, University of Paris-Saclay, Jouy-en-Josas, France; ^2^Systems and Synthetic Biology Lab, CEA, CNRS, UMR 8030, Genomics Metabolics, University Paris-Saclay, Évry, France; ^3^SYNBIOCHEM Center, School of Chemistry, Manchester Institute of Biotechnology, University of Manchester, Manchester, United Kingdom

**Keywords:** mathematical model, cell-free, prototyping, resource competition, transcription, translation, metabolism

## Abstract

Cell-free TX-TL is an increasingly mature and useful platform for prototyping, testing, and engineering biological parts and systems. However, to fully accomplish the promises of synthetic biology, mathematical models are required to facilitate the design and predict the behavior of biological components in cell-free extracts. We review here the latest models accounting for transcription, translation, competition, and depletion of resources as well as genome scale models for lysate-based cell-free TX-TL systems, including their current limitations. These models will have to find ways to account for batch-to-batch variability before being quantitatively predictive in cell-free lysate-based platforms.

## Introduction

All the processes required to produce proteins in bacteria can be performed by adding DNA to a cell-free platform. After lysis of living cells, transcription, translation, degradation, and protein folding continue to operate as they do *in vivo* (Hodgman and Jewett, 2012; Sun et al., 2013; Takahashi et al., 2015a). Metabolic pathways like glycolysis or pentose phosphate pathway remain active and are used to regenerate ATP and maximize protein production over time (Kim and Swartz, 2001; Calhoun and Swartz, 2005). Protein production outside of the cell simplifies gene expression with well-defined parameters, easy to control inputs, faster time scale, and less numerous unknown interactions. As a result, many laboratories use cell-free as a prototyping platform to characterize expression of single proteins or complex metabolic pathways (Takahashi et al., 2015b; Wu et al., 2017; Borkowski et al., 2018). Mathematical models dedicated to cell-free emerged to predict protein production and understand the limits of this new platform. Cell-free properties are close to living organisms as the same processes take place in both systems, yet significant differences exist. For example, molecular crowding (Spruijt et al., 2014) and resources distribution (Sun et al., 2013) are significantly altered in cell-free and there is no resource competition with the host. Such differences oblige synthetic biologists to adapt the models already developed for living cells. This short review focuses on the recent deterministic models developed to understand lysate-based cell-free platforms and used to predict the behavior of simple or complex pathways (Table 1). Those models pave the way for efficient metabolic engineering in the emerging field of cell-free synthetic biology.

**Table 1:** Deterministic models developed to understand cell-free.

**Modeling strategy**	**Problems tackled**	**Level of detail**	**Description**	**Strength**	**Weakness**	**References**
ODE	Protein production in cell-free	Simple^*^	Michaelis-Menten for the translation processes, as well as for degradation	Simple, quantitative	Parameters values are experiment dependent	Karzbrun et al., 2011
ODE	Protein production in cell-free	Simple^*^	Simple description of the transcription and translation processes. One parameter summarize each	Simple, qualitative	Parameters values are experiment dependent	Stögbauer et al., 2012; Siegal-Gaskins et al., 2014
ODE	Protein production in cell-free Resource competition in cell-free	Complex^*^	Detailed modeling: Particular focus on the translation process and the competition for the translation machinery	Generalization power quantitative	Parameters values are experiment dependent	Underwood et al., 2005; Borkowski et al., 2018
ODE	Protein production in cell-free Resource competition in cell-free	Simple^*^	Binding, unbinding and elongation bundled in one parameter. Accounts for number of RNAP, ribosomes, and promoter/RBS strengths	Easily adaptable to a new situation or phenomena	Necessitates parameter determination for new situation	Gyorgy and Murray, 2016; Halter et al., 2018; Voyvodic et al., 2018
ODE	Protein production in cell-free Resource depletion in cell-free	Complex^*^	Accounts for all known processes, including NTP consumption and degradation	Models biochemical phenonema precisely so generalisable	Important parameter identification/estimation needed	Siegal-Gaskins et al., 2013; Tuza et al., 2013; Nieß et al., 2017; Matsuura et al., 2018; Moore et al., 2018
Constraint based	Protein production with cell-free Metabolism in cell-free	Complex^*^	Modification of *E. Coli* metabolic model to account for cell-free constraints	Accounts for the full metabolism, can use constraints based methods such as FBA	Defining the objectives, require deeper knowledge of reactions in cell-free	Vilkhovoy et al., 2018

* *Simple stands for one protein produced and limited amount of parameters (< 10) Complex stands for more than one protein produced or/and large amount of parameters (more than 10)*.

## Translation and transcription processes in cell-free

**Figure 1:**
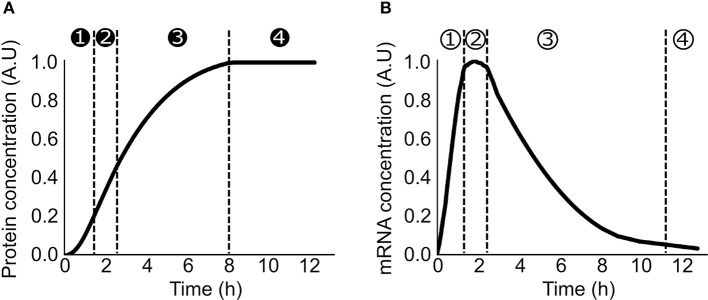
Production of a constitutively expressed gene in cell-free **(A)** Protein concentration over time in a cell-free platform. ➊ the production rate increase over time ➋ the production rate is constant ➌ the production rate decreases ➍ the production rate is null **(B)** mRNA concentration over time in a cell-free platform ① the concentration increases over time ② the concentration is constant ③ the concentration decreases over time ④ the concentration is close to zero.

Lysate-based cell-free consists of a crude cell extract supplemented with buffer, amino acids, NTP, NAD, PEG, tRNA, and metabolic intermediates (Sun et al., 2013). A major advantage of cell-free is the absence of host regulations (Hodgman and Jewett, 2012), allowing circuits to function in isolation and an easy quantitative description of gene expression. A constitutively expressed gene in cell-free exhibits specific patterns at the translation and transcription levels. Protein production can be divided in 4 phases: in phase 1, the production rate increases over time, in phase 2, the production rate is constant during a 30 min/1 h, in phase 3 the production rate decreases slowly and eventually in phase 4 the production rate is null (Siegal-Gaskins et al., 2014; Figure 1A). A similar 4 phases pattern is observed with the mRNA concentration (Siegal-Gaskins et al., 2014; Figure 1B). ODE Models describing transcription, translation, mRNA, and protein degradation processes at various scales have been successfully used to predict mRNA and protein dynamics in lysate-based systems (Stögbauer et al., 2012; Tuza et al., 2013; Siegal-Gaskins et al., 2014; Nieß et al., 2017; Borkowski et al., 2018; Matsuura et al., 2018; Moore et al., 2018). DNA concentrations are usually considered constant: degradation is neglected as plasmid DNA or protected linear template are used, and replication is considered not to happen since no dNTPs are added to the reaction mix.

Numerous models, with varying degrees of complexity, try to reproduce those production phases observed in cell-free reactions.

A simple model based on only 4 reactions and 10 parameters is sufficient to fit the full mRNA and protein dynamics during the first hours of reaction (Karzbrun et al., 2011). The transcription process is reduced to one step in which the RNA polymerase binds to the DNA; the rate of mRNA production depends only this binding rate and the DNA length. Similarly, the translation process is described as one binding step of the ribosome on the mRNA with the rate of protein production depending only on the binding rate and the mRNA length. This model is appropriate for the first hours before the consumption of resources and/or the waste accumulation (e.g., ATP degradation, toxic metabolites…) cause the reaction to stop (Siegal-Gaskins et al., 2014). A simple way to simulate the slow decay in synthesis is the consumption of the NTP over time. The transcription reaction slows down and eventually stops (Stögbauer et al., 2012; Tuza et al., 2013). The decrease in the NTP concentration (Kim and Swartz, 2001; Jewett et al., 2008; Moore et al., 2018) is an efficient method to obtain a decreasing transcription over time and simulate protein and mRNA production in cell-free but no experimental data either confirms or denies this approach. The accumulation of inactive RNA polymerase/ribosome (Failmezger et al., 2016; Moore et al., 2018), accumulation of toxic metabolites (Kim and Swartz, 2000), or increase of the relative RNases concentration compare to the total amount of mRNA (Siegal-Gaskins et al., 2014) are possible other explanations of the arrest of protein production after 8 h. Voyvodic et al. (2018) added terms corresponding to decreasing resources for protein production and accumulation of toxic by-products as a reduction in production rates parametrized by a Michaelis-Menten like ratio, as an elegant way to account for the slowing production rate. As all cell-free models trying to account for the end of production after 8 h, the main issue is identifying the exact cause for decreasing production.

Models using Michaelis–Menten kinetics also succeed to capture protein production pattern in cell-free (Stögbauer et al., 2012). Those models precisely captured the observable mRNA and proteins dynamics in cell-free while remaining relatively coarse-grained. The model of Tuza et al. add extra steps in the transcription (and translation) process with a reversible binding of the RNA polymerase (ribosome) on the DNA (mRNA) followed by a reversible binding of the first NTP (amino acid) and eventually an irreversible elongation step. Moore et al. also developed a model accounting for reversible binding, unbinding and elongation steps, sharing the NTP energy source. Those more detailed descriptions of the transcription and translation processes lead to accurate predictions of the data obtained in cell-free and capture additional properties (Stögbauer et al., 2012; Tuza et al., 2013; Borkowski et al., 2018; Moore et al., 2018). For example, the non-additive cost of protein production when several genes are expressed requires higher level of complexity to be predicted (Borkowski et al., 2018).

While all models presented in this section described transcription and translation processes, the main challenge they faced is proper parameter identification, as biochemical parameters can vary widely from batch to batch and from *in vivo* to cell-free systems. Currently, models often used components concentration measured *in vivo* and estimated their concentration based on the dilution factor of the *E. coli* cytoplasm after the lysate extraction protocol, which is not entirely satisfactory.

## Resource competition in cell-free

Resource competition is an important phenomenon that impacts circuit behavior in cell-free systems and should be accounted for in modeling approaches.

**Figure 2:**
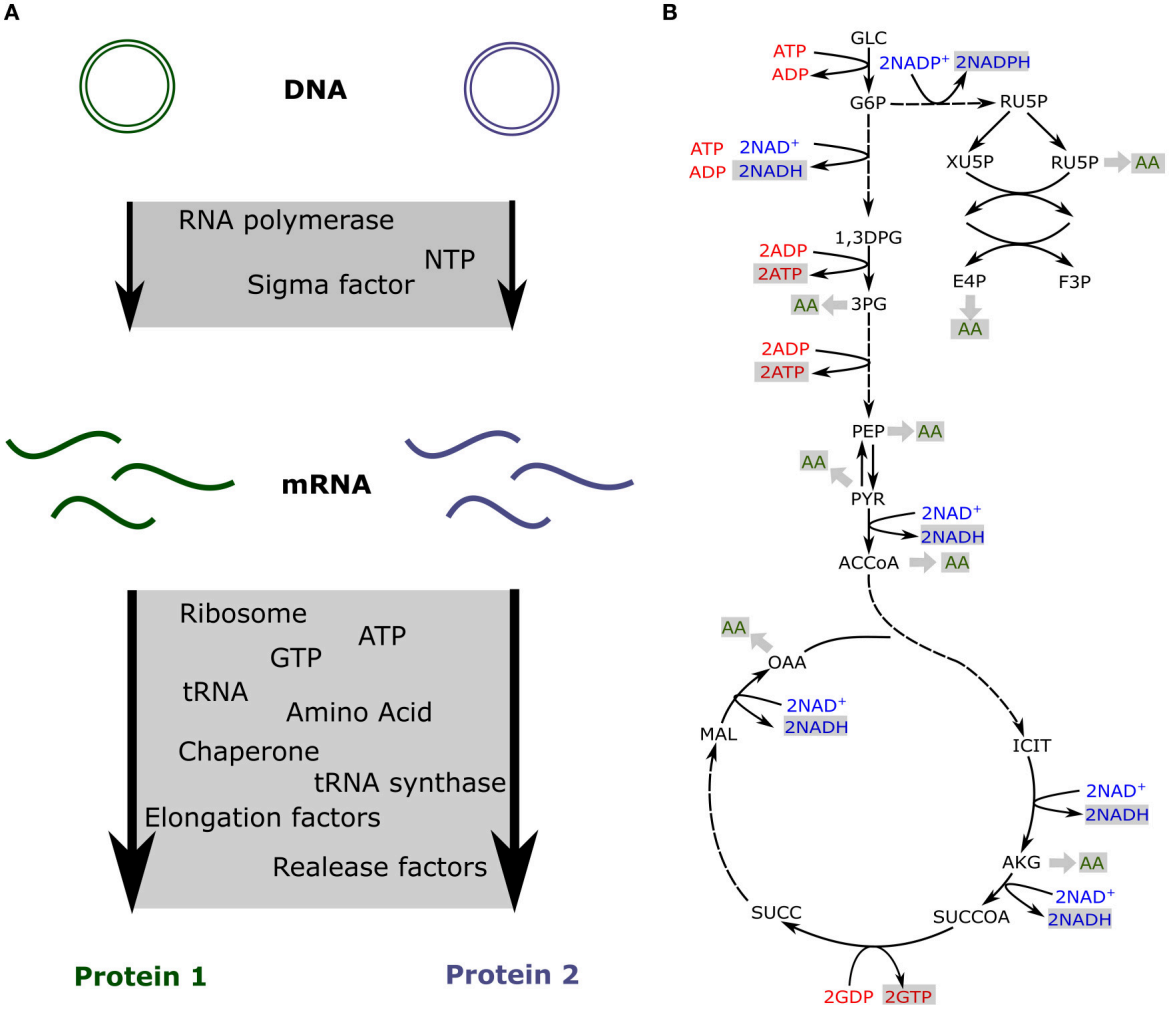
Resource competition in cell-free **(A)** Competition for resources between two genes expressed in cell-free. The transcription and translation machineries amounts are fixed **(B)** Production of energy and amino acids via the core metabolic network describing glycolysis, pentose phosphate pathway, the TCA cycle and the Entner-Doudoroff pathway.

As a fixed amount of resources is present in the cell-free extract, competition has been measured between synthetic circuits (Siegal-Gaskins et al., 2014; Borkowski et al., 2018; Moore et al., 2018). Some of the previously described models take into account the limitation of each resource and include a fixed amount of transcription and translation machineries to predict the impact of resource competition in cell-free (Figure 2A; Siegal-Gaskins et al., 2013; Borkowski et al., 2018; Moore et al., 2018).

A maximal protein production is measured after a few hours before resources depletion and degradation (Figure 1). This upper limit on production rate is the result of one or several limited resources (RNA polymerase, NTP, ribosome, elongation factors, amino acids, chaperone, tRNA synthetase, or tRNA). DNA, NTP, amino acids, and T7 RNA polymerase are directly added to the mix so their impacts on the protein production can be easily measured. Increasing DNA concentration leads to an increase of protein production until a saturation point is reached (Siegal-Gaskins et al., 2014; Borkowski et al., 2018; Voyvodic et al., 2018), and toxicity can be observed with high DNA concentration (Borkowski et al., 2018). T7 polymerase (Siegal-Gaskins et al., 2014), amino acids, tRNA and nucleotides (Shin and Noireaux, 2010) are present in excess in the cell-free mix causing no noticeable competition for these resources. Eventually, High NTP concentration negatively affects the translation process (Nagaraj et al., 2017). Natural transcription and translation machineries are less controlled as they are added via the crude extract. Indirect measurements using competition for resources between two plasmids are used to deduce competition for transcription and translation machineries (Underwood et al., 2005; Siegal-Gaskins et al., 2014; Gyorgy and Murray, 2016; Borkowski et al., 2018; Moore et al., 2018). The main source of competition can be at the transcription and/or translation level depending of the extract and the level of protein produced (Underwood et al., 2005; Li et al., 2014; Siegal-Gaskins et al., 2014; Gyorgy and Murray, 2016; Borkowski et al., 2018; Moore et al., 2018; Voyvodic et al., 2018). Parametrization of the models using the appropriate RNA polymerase and ribosomes concentrations and binding/unbinding rates allows an accurate description of the resource competition and to fit properly the production of several proteins expressed concurrently in cell-free (Siegal-Gaskins et al., 2014; Borkowski et al., 2018; Moore et al., 2018). Accounting for RNAP and ribosomes sharing between parts can also be leveraged to minimize the number of experiments required to fit parameters and obtain a predictive model (Halter et al., 2018). While not the main focus of this review, parameter estimation or identification is a major hurdle of detailed models, and techniques from systems biology (e.g., Lillacci and Khammash, 2010) can be used to tackle this issue.

The models presented in this sections, while being able to account for transcription, translation and resource competition from a lack of generalizability due both to variability in experimental conditions as batches can differ greatly, and to scarcity of biochemical work measuring those parameters in cell-free setting as has been estimated from *in vivo* measurements.

## Metabolism in cell-free

The models presented in this section are constraint based, so as to take the whole metabolism into account and not the circuit in isolation as done in the previous sections.

In cell-free platforms, translation, and transcription are not the only active processes. Glycolysis, pentose phosphate pathway, TCA cycle, Entner-Doudoroff pathway, and amino acid biosynthesis are still producing ATP, reducing equivalents, and amino acids (Kim and Swartz, 2001; Jewett and Swartz, 2004a,b). The previously described models account for the resources competition for a fixed amount of transcription and translation resources and usually do not include any metabolite production or consumption. Such an approach is quite limiting for metabolic pathway prototyping as those circuits also compete for metabolites (Wu et al., 2017; Borkowski et al., 2018). Constraint-based models have been used to simulate metabolites production and consumption when various proteins are produced at different levels (Vilkhovoy et al., 2018; Figure 2B). This model coupled transcription and translation processes with the availability of metabolic resources. Flux balance analysis was adapted to cell-free conditions with the objective function being the maximization of the protein translation rate. Growth associated reactions were removed and cell-free specific deletions were added from *E. coli* metabolic model, leading to 264 reactions and 146 species (Vilkhovoy et al., 2018). The stoichiometric network was adjusted to cell-free and fluxes were constrained by experimental measurements of glucose, nucleotides, amino and organic acid consumption and production rates. The transcription and translation were bound by Michaelis-Menten formula with a maximum transcription and translation rate depending on RNA polymerase concentration, RNA polymerase elongation rate, gene length, promoter strength, and the ribosome concentration, a polysome amplification constant, the ribosome elongation rate, the protein length, and the RBS strength, respectively. The energy efficiency was calculated using the ATP cost by transcription and translation processes. Transcription and translation rates are subject to resource constraints encoded by the metabolic network (Figure 2B). This model efficiently predicts proteins production and simulates optimal flux distribution in cell-free metabolic network. It makes predictions possible for metabolic engineering in cell-free as metabolites produced or consumed by a pathway will be accounted for via its energy efficiency.

Constraint based modeling for cell-free systems is an interesting field that would need further developments from the research community, both to include cell-free specific constraints and reactions, as well as to account for dynamic behavior such as metabolite exhaustion in cell-free systems.

## Conclusion

Cell-free appeared as the ideal platform for circuits prototyping. It accelerates characterization and avoids the impact of the host on the circuit behavior. Models can be easily parametrized and predictions are easier and more accurate than *in vivo*, for qualitative behavior. Parametrization for quantitative behavior can be tackled using techniques from systems biology. Simple models succeed to accurately predict the simultaneous production levels of multiple proteins and the competition for the limited amount of resources in cell-free. A certain level of complexity is necessary to capture competition for metabolites but produces a powerful tool for metabolic engineering. The main limit for lysate-based cell-free in metabolic engineering and modeling remains extract preparation: extract efficiency can differ strongly depending on the experimentalist leading to variability of protein production and necessity of robust controls for each new batch, as well as uncertain parameters that vary with each batch for the modeler. Preliminary control of the extract quality and tuning of model parameters on each batch is required to obtain accurate predictions and precludes generalization. A way forward to both increase reproducibility and predictive modeling in cell-free systems would be a higher degree of automation in the extract production providing robust lysate preparation at affordable price.

## Author contributions

All authors listed have made a substantial, direct and intellectual contribution to the work, and approved it for publication.

### Conflict of interest statement

The authors declare that the research was conducted in the absence of any commercial or financial relationships that could be construed as a potential conflict of interest.
